# Heteronanowires of MoC–Mo_2_C as efficient electrocatalysts for hydrogen evolution reaction†
†Electronic supplementary information (ESI) available: SEM, XRD, FT-IR, TG/DSC and CHN elemental analysis of hybrid precursors, additional XPS results, SEM, TEM, N_2_-sorption isothermals, Raman spectra, CV and EIS of a series of MoC_*x*_ and their comparison with previously reported noble-metal free catalysts. See DOI: 10.1039/c6sc00077k


**DOI:** 10.1039/c6sc00077k

**Published:** 2016-02-12

**Authors:** Huanlei Lin, Zhangping Shi, Sina He, Xiang Yu, Sinong Wang, Qingsheng Gao, Yi Tang

**Affiliations:** a Department of Chemistry , Jinan University , Guangzhou 510632 , China . Email: tqsgao@jnu.edu.cn; b Department of Chemistry , Shanghai Key Laboratory of Molecular Catalysis and Innovative Materials , Laboratory of Advanced Materials and Collaborative Innovation Center of Chemistry for Energy Materials , Fudan University , Shanghai 200433 , China . Email: yitang@fudan.edu.cn; c Analytic and Testing Centre , Jinan University , Guangzhou 510632 , China

## Abstract

Exploring efficient noble-metal free electrocatalysts for the hydrogen evolution reaction (HER) is one of the most promising pathways for facing the energy crisis. Herein, MoC–Mo_2_C heteronanowires composed of well-defined nanoparticles were accomplished *via* controlled carbonization, showing excellent HER activity, fast kinetic metrics and outstanding stability in both acid and basic electrolytes. In particular, the optimal one consisting of 31.4 wt% MoC displayed a low overpotential (*η*_10_ = 126 and 120 mV for reaching a current density of –10 mA cm^–2^), a small Tafel slope (43 and 42 mV dec^–1^) and a low onset overpotential (38 and 33 mV) in 0.5 M H_2_SO_4_ and 1.0 M KOH, respectively. Such prominent performance, outperforming most of the current noble-metal free electrocatalysts, was ascribed to the carbide surface with an optimized electron density, and the consequently facilitated HER kinetics. This work elucidates a feasible way toward efficient electrocatalysts *via* heteronanostructure engineering, shedding some light on the exploration and optimization of catalysts in energy chemistry.

## Abstract 2

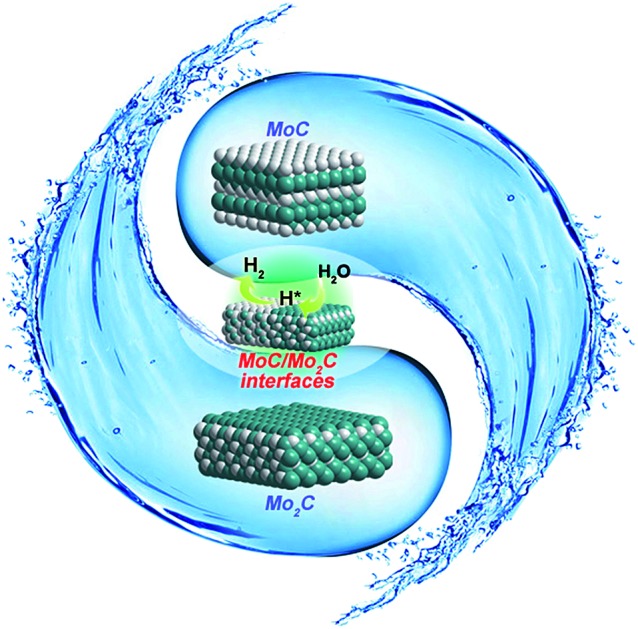
MoC–Mo_2_C heteronanowires accomplished *via* controlled carbonization are efficient in the hydrogen evolution reaction due to a synergistic enhancement.

## Introduction

The rapid growth of global energy consumption and the associated environmental issues have triggered an urgent demand for renewable and clean energy sources.^1^,^2^ Hydrogen (H_2_) is a promising candidate as it stores energy from renewable sources (*e.g.*, sunlight and wind) into the chemical bond *via* the electrolysis of water, which then can be released through the reverse reaction in fuel cells on demand.^3^ The hydrogen evolution reaction (HER) *via* water electrolysis essentially depends on the efficiency of electrocatalysts, which must be stable and capable of reducing water rapidly at potentials close to its thermodynamic value.^4^,^5^ Although noble metals, *e.g.*, platinum, show high activity, they are severely limited by their high cost and low abundance.^6^,^7^ It is urgently demanded to develop noble-metal free catalysts with good activity, long-term stability, high element-abundance, and economical cost.^5^,^8^–^10^

Remarkable advances have been recently made regarding the use of transition-metals and their carbides, nitrides, chalcogenides and phosphides.^5^,^8^,^10^,^11^ Presenting varied electronic features and catalytic properties related to tunable phases and composition,^12^–^15^ molybdenum carbides (MoC_*x*_) have received special attention as one of the promising noble-metal free catalysts. Among them, Mo_2_C demonstrates the best performance because of its electron configuration around the Fermi level (*E*_F_).^13^,^16^ Intense effort has been devoted to Mo_2_C nanostructures with enriched active-sites,^17^–^26^ and composites integrating a conducting matrix, *e.g.*, carbon nanotubes (CNTs) and graphene (GR).^27^–^31^ However, the negative hydrogen-binding energy (Δ*G*_H*_) on Mo_2_C indicates a strong adsorption of H on the Mo_2_C surface, which benefits H^+^ reduction (*i.e.*, Volmer step), but restricts H_ads_ desorption (*i.e.*, the Heyrovsky/Tafel step).^16^,^32^ Thus, an optimization of the electronic features are desired. The introduction of doping elements has even been employed,^33^,^34^ *via* which the improvement is however limited due to inadequate modification and inevitable structure damage. It is notable that the electron density around the Mo active-sites mostly relies on the carbon in the lattice,^35^,^36^ which will be reduced with increasing C because of the electron-transfer from Mo to C.^14^ For example, with a high C content, MoC usually presents weaker hydrogen binding in comparison with that on Mo_2_C, and consequently a facilitated Heyrovsky/Tafel step, but a hindered Volmer reaction.^13^,^29^ Regarding the respectively promoted elementary reactions of HER on Mo_2_C and MoC, it's promising to achieve a synergistically-enhanced activity on MoC–Mo_2_C interfaces, which are rarely reported to the best of our knowledge.

Herein, we report novel MoC–Mo_2_C heteronanowires (HNWs) as efficient HER electrocatalysts, which are fabricated from MoO_*x*_–amine nanowires (NWs) *via* controlled carbonization. The HNWs denoted as MoC–Mo_2_C-*n* (where *n* refers to the MoC weight percentage) are one-dimensional (1D) heterostructures composed of defined nanoparticles (NPs), with rich nanoporosity, large surface area, and more importantly a tunable composition. This is remarkably improved from our previous work on nanoporous Mo_2_C,^21^ highlighted by the effective electron regulation and further improved activity *via* varying MoC/Mo_2_C in the HNWs. With an optimal composition, MoC–Mo_2_C-31.4 exhibits a low *η*_10_ (overpotential required to reach a current density of –10 mA cm^–2^) of 126 mV, a low Tafel slope of 43 mV dec^–1^, and a low *η*_onset_ (overpotential referring to the beginning of the linear regime in the Tafel plot) of 38 mV in 0.5 M H_2_SO_4_, outperforming most of the current noble-metal free electrocatalysts. The high HER activity should be ascribed to the moderated electron density on the carbide surface, which optimizes the hydrogen-binding and thus the HER kinetics. In addition, the good efficiency in basic electrolyte further verifies MoC–Mo_2_C HNWs as promising noble-metal free electrocatalysts.

## Results and discussion

**Fig. 1:**
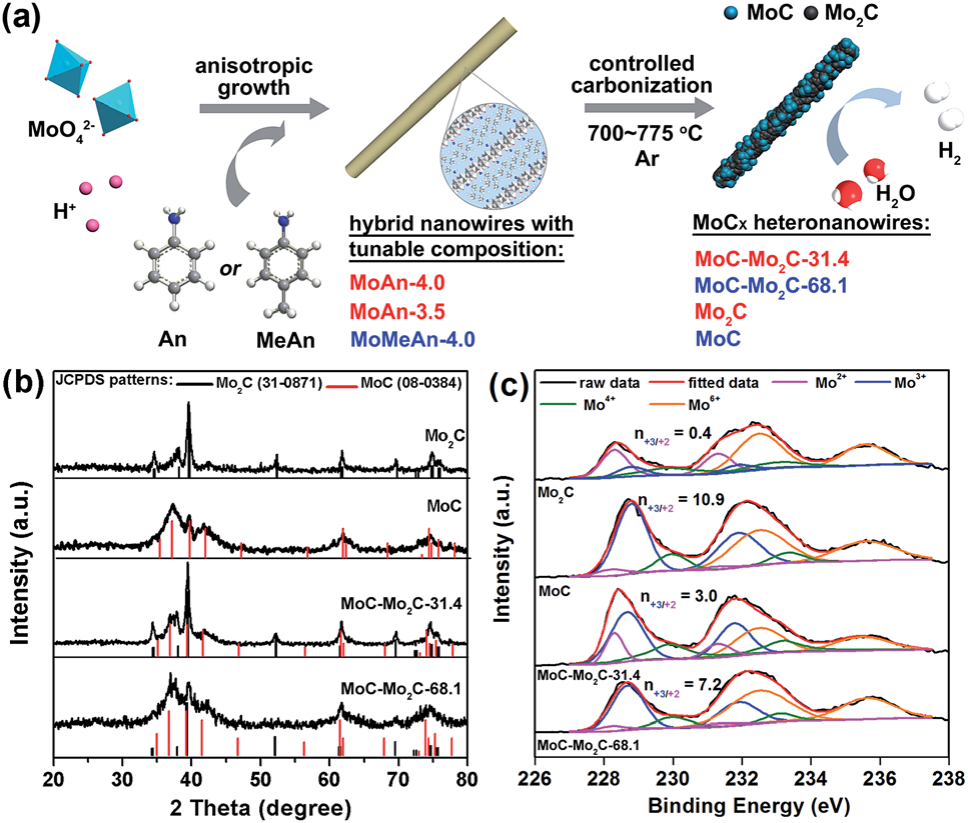
(a) Schematic illustration for the fabrication of MoC_*x*_ HNWs from MoO_*x*_–amine NWs with tunable composition. (b) XRD patterns and (c) Mo 3d XPS profiles of the as-obtained MoC_*x*_ NWs.

As shown in Fig. 1a, a series of MoC_*x*_ HNWs can be achieved *via* the controlled carbonization of various MoO_*x*_–amine precursors (Table S1†), which were firstly fabricated through reacting molybdate with aniline (An) or *p*-methylaniline (MeAn). The wire-like precursors (Fig. S1†) obtained with An at pH 4.0 (MoAn-4.0) and 3.5 (MoAn-3.5) were respectively confirmed as Mo_3_O_10_(C_6_H_8_N)_2_·2H_2_O (JCPDS no. 50-2402) and its mixture with Mo_8_O_25_(C_6_H_8_N)_2_·2H_2_O (JCPDS no. 49-2068), using X-ray diffraction (XRD, Fig. S2†). For those obtained with MeAn (MoMeAn-4.0), a similar XRD pattern with an obvious shift to lower degree values suggests an analogous crystalline structure with an expanded lattice due to the large MeAn molecule. Their composition was further evidenced using Fourier transform infrared (FT-IR), thermogravimetric analysis coupled with differential scanning calorimetry (TGA/DSC), and CHN elemental analysis (Fig. S3†). Obviously, the different carbon content will benefit the controlled synthesis of MoC_*x*_.^37^

The XRD investigation clearly confirms the achievement of various MoC_*x*_ (Fig. 1b), whose composition was further determined through the combined measurements of XRD, CHN elemental analysis and inductively coupled plasma (ICP) (Table S2†). The product α-Mo_2_C (JCPDS no. 31-0871) was obtained from calcining MoAn-3.5 at 775 °C, and η-MoC (JCPDS no. 08-0384) was obtained from MoMeAn-4.0 at 700 °C. The heterostructures of MoC–Mo_2_C-31.4 and MoC–Mo_2_C-68.1 were harvested from MoAn-4.0 and MoMeAn-4.0, respectively, at 775 °C. As expected, the carbon source in the hybrid precursors contributes to the tailored generation of carbides. The higher carbon content of MoAn-4.0 (22.9%) compared to that of MoAn-3.5 (20.7%) benefits the formation of some MoC in Mo_2_C, and having sufficient carbon (25.3%) in MoMeAn-4.0 leads to the pure phase of MoC.

These samples were further analyzed using X-ray photoelectron spectroscopy (XPS, Fig. 1c). The peak fitting of Mo 3d profiles suggests that there are four states (+2, +3, +4 and +6) for Mo on the surface.^17^,^38^ The Mo^4+^ and Mo^6+^ species result from the inactive MoO_2_ and MoO_3_, respectively, which are commonly observed as carbides are exposed to air.^39^ We focus on the Mo^2+^ and Mo^3+^ species with peaks at 228.0–229.0 eV (Mo 3d_5/2_) and 231.0–232.0 eV (Mo 3d_3/2_), because they are the active centres for electrocatalytic HER.^13^,^17^ The Mo^3+^/Mo^2+^ mole ratios (*n*_3+/2+_) on the MoC_*x*_ surface can provide useful information to understand the nature of the active-sites (Table S3†). The *n*_3+/2+_ values for Mo_2_C and MoC are 0.4 and 10.9 (Fig. 1c), respectively, which suggests that Mo^2+^ is dominant to Mo^3+^ on Mo_2_C, while Mo^3+^ is prevailing on MoC. In the heterostructures, *n*_3+/2+_ visibly changed to 3.0 for MoC–Mo_2_C-31.4, and 7.2 for MoC–Mo_2_C-68.1. Such a variation of Mo^3+^/Mo^2+^ will influence the HER activity, related to the different electron density around Mo^3+^ and Mo^2+^.^13^

Meanwhile, the Raman spectra of the above MoC_*x*_ samples display the D- and G-bands of carbon at 1350 and 1590 cm^–1^, respectively, confirming the presence of free carbon (Fig. S4†).^25^ In addition, N_2_ isothermal sorption reveals the large surface of the MoC_*x*_ NWs (Fig. S5†). Particularly, MoC–Mo_2_C-31.4 HNWs present a specific surface area of 58.5 m^2^ g^–1^, larger than that of Mo_2_C (39.3 m^2^ g^–1^), MoC–Mo_2_C-68.1 (33.7 m^2^ g^–1^) and MoC (26.0 m^2^ g^–1^). A major pore distribution at around 5.5 nm is observed for Mo_2_C and MoC–Mo_2_C-31.4.

**Fig. 2:**
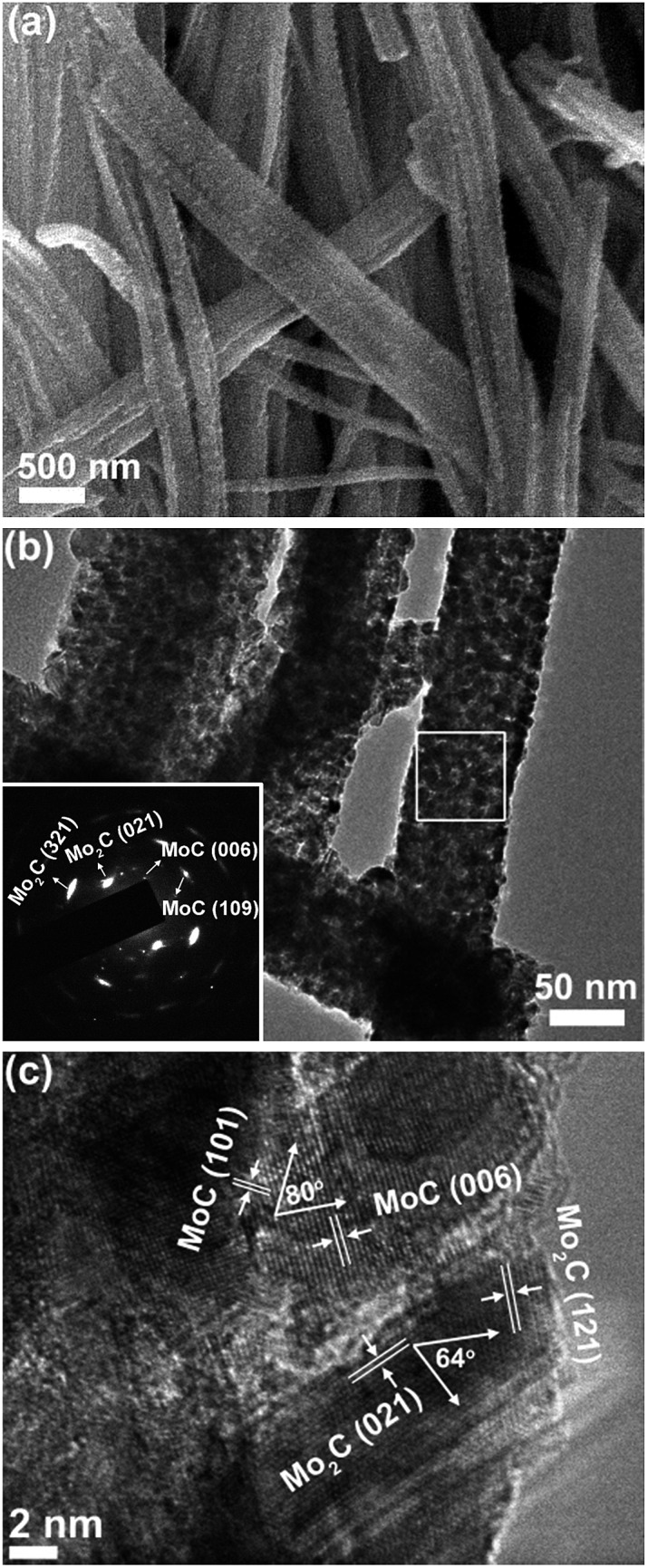
(a) SEM, (b) TEM and (c) HR-TEM images of MoC–Mo_2_C-31.4 HNWs. Inset of (b) is the SAED pattern obtained on the marked area.

Taking MoC–Mo_2_C-31.4 as the model sample, the heteronanowires can be well confirmed using scanning electron microscopy (SEM) and transmission electron microscopy (TEM). Wire-like products several micrometres in length and 80–150 nm in width are observed in Fig. 2a, maintaining the 1D morphology of the precursors. TEM further displays that such NWs are composed of NPs (∼10 nm), and the selected area electron diffraction (SAED) pattern corresponds to those of α-Mo_2_C and η-MoC (Fig. 2b). Accordingly, the (121) and (021) lattice fringes of α-Mo_2_C and the (101) and (006) fringes of η-MoC are identified in the high resolution TEM (HR-TEM, Fig. 2c). Noticeably, the interfaces between close-stacking MoC and Mo_2_C NPs are visible, which would benefit synergy of the surface activity. Analogously, the Mo_2_C and MoC NWs composed of the corresponding NPs are also verified through the TEM investigation, and in the MoC–Mo_2_C-68.1 HNWs, both the MoC and Mo_2_C NPs are identified (Fig. S6†). With nanosized crystallites, enriched nanoporosity, large surface areas, a conducting carbon matrix, and more importantly tunable Mo^3+^/Mo^2+^ centres, MoC–Mo_2_C HNWs are expected to efficiently catalyse the HER.

**Fig. 3:**
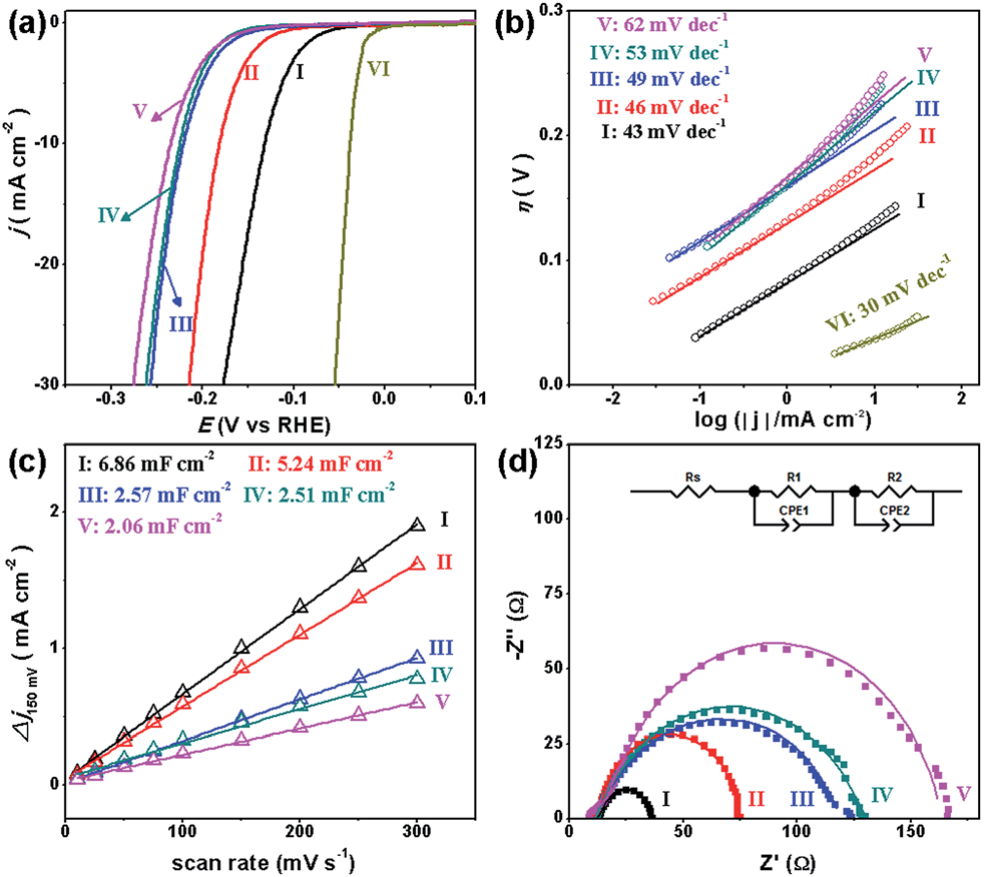
(a) Polarization curves and (b) Tafel plots for the HER on modified GCEs comprising (I) MoC–Mo_2_C-31.4, (II) Mo_2_C, (III) MoC–Mo_2_C-68.1, (IV) MoC–Mo_2_C-30 (mixed), (V) MoC, and (VI) commercial Pt/C in 0.5 M H_2_SO_4_. (c) Estimation of *C*_dl_ through plotting the current density variation (Δ*j* = (*j*_a_ – *j*_c_)/2, at 150 mV *vs.* RHE; data obtained from the CV in Fig. S7†) against scan rate to fit a linear regression, and (d) Nyquist plots (at *η* = 200 mV) of the above MoC_*x*_ electrocatalysts.

To investigate the HER performance in an acidic electrolyte, the as-prepared MoC_*x*_ NWs were loaded onto glassy carbon electrodes (GCEs) with a mass loading of 0.14 mg cm^–2^. Fig. 3a displays their polarization curves with *iR*-drop corrections in 0.5 M H_2_SO_4_, along with that of the benchmark Pt/C catalyst (40 wt% Pt on carbon black from Johnson Matthey) for reference. Among the MoC_*x*_ catalysts, MoC–Mo_2_C-31.4 exhibits the highest activity. To achieve a current density (*j*) of –10 mA cm^–2^, MoC–Mo_2_C-31.4 requires a *η*_10_ of 126 mV, which is obviously lower than those of α-Mo_2_C (182 mV) and η-MoC (232 mV). This suggests a synergic enhancement between Mo_2_C and MoC in the HNWs. Meanwhile, the mechanically-mixed MoC–Mo_2_C NWs with a similar MoC content of 30 wt% (denoted as MoC–Mo_2_C-30 (mixed)) displayed a lower activity (*η*_10_ = 222 mV), indicating that the MoC–Mo_2_C interfaces on the nanoscale in MoC–Mo_2_C-31.4 contribute to the efficient HER. Such a synergic effect is prohibited by the high percentage of MoC in the HNWs, and as this was increased to 68.1%, the activity obviously reduced. A summary of the HER activity of the above MoC_*x*_ is listed in Table 1.

**Table 1:** Summary of the HER activity of MoC–Mo_2_C-31.4, Mo_2_C, MoC–Mo_2_C-68.1, MoC–Mo_2_C-30 (mixed), and MoC in 0.5 M H_2_SO_4_

Cat.	*η*_10_ (mV)	*η*_onset_ (mV)	Tafel slope (mV dec^–1^)	*R*_ct_ ^*a*^ (Ω)	*C*_dl_ ^*b*^ (mF cm^–2^)	*j*_0_ ^*c*^ (mA cm^–2^)
MoC–Mo_2_C-31.4	126	38	43	21.8	6.86	1.1 × 10^–2^
Mo_2_C	182	61	46	60.4	5.24	1.2 × 10^–3^
MoC–Mo_2_C-68.1	218	65	52	101.7	2.57	7.2 × 10^–4^
MoC–Mo_2_C-30 (mixed)	222	100	53	116	2.51	3.2 × 10^–4^
MoC	232	105	62	145	2.06	5.0 × 10^–4^

^*a*^Data was measured at *η* = 200 mV.

^*b*^Data was calculated according to the CV results (Fig. S7).

^*c*^Exchange current densities (*j*_0_) were obtained from Tafel curves by using extrapolation methods.

Accordingly, the Tafel plots of the above carbides present the same order in HER kinetics (Fig. 3b and Table 1). Among them, MoC–Mo_2_C-31.4 shows a *η*_onset_ of 38 mV and a Tafel slope of 43 mV dec^–1^, which are obviously lower than those of Mo_2_C, MoC, MoC–Mo_2_C-68.1 and MoC–Mo_2_C-30.0 (mixed). The small Tafel slope of MoC–Mo_2_C-31.4 indicates a fast increase of the hydrogen generation rate with the applied overpotential, corresponding to the high activity presented in the polarization curves. According to the classic theory, the HER in acidic aqueous media proceeds in two steps (eqn (1)–(3)),^40^,^41^ where the * indicates the active site, and H* is a hydrogen atom bound to an active site. The first one is an electrochemical reduction step (H^+^ reduction, Volmer-reaction) with a Tafel slope of 118 mV dec^–1^ (eqn (1)), and the second one (H_ads_ desorption) is either the ion and atom reaction (Heyrovsky-reaction) with a slope of 40 mV dec^–1^ (eqn (2)) or the atom combination reaction (Tafel-reaction) with a slope of 30 mV dec^–1^ (eqn (3)).^8^,^40^,^41^ Although the Tafel slope alone is insufficient to determine the specific mechanism, the evidently reduced slope for MoC–Mo_2_C-31.4, compared with MoC and MoC–Mo_2_C-68.1, still confirms the promoted Volmer-step in the HER kinetics.^42^,^43^ In addition, the exchange current density (*j*_0_) of the above electrocatalysts was further calculated by extrapolating the Tafel plots, which is the most inherent measure of HER activity. As expected, the *j*_0_ of 1.1 × 10^–2^ mA cm^–2^ for MoC–Mo_2_C-31.4 is higher than that of the other MoC_*x*_ (Table 1).

1 H_3_O^+^(a.q.) + e^–^ + * → H* + H_2_O(l)

2 H_3_O^+^(a.q.) + e^–^ + H* → * + H_2_(g) + H_2_O(l)

3 H* + H* → 2* + H_2_(g)

The electrochemical surface area (ECSA) and resistant charge-transfer (*R*_ct_) were further evaluated to provide insight into the MoC_*x*_ electrocatalysts (Table 1, Fig. 3c and d). Although the accurate measurement of ECSA is difficult owing to the unclear capacitive behaviour, it can be visualized through calculating the double-layer capacitances (*C*_dl_) which are proportional to the ECSA values.^44^ An estimation of *C*_dl_ using the cyclic voltammograms (CV, Fig. S7†) in 0.5 M H_2_SO_4_ were alternatively utilized to provide a relative comparison.^25^,^45^ As shown in Fig. 3c, the *C*_dl_ of 6.86 mF cm^–2^ presented by MoC–Mo_2_C-31.4 is higher than those on α-Mo_2_C (5.24 mF cm^–2^), η-MoC (2.06 mF cm^–2^), MoC–Mo_2_C-68.1 (2.57 mF cm^–2^), and MoC–Mo_2_C-30 (mixed) (2.51 mF cm^–2^). Regarding the *C*_dl_ associated with the active surface area, the current density divided by *C*_dl_ can further reflect the intrinsic activity,^46^,^47^ from which the remarkably high one for MoC–Mo_2_C-31.4 indicates intrinsic optimization of the active-sites (Fig. S8†). Meanwhile, their electrochemical impedance spectroscopy (EIS) measurements show the consistent order in *R*_ct_, and a *R*_ct_ as low as 21.8 Ω delivered by MoC–Mo_2_C-31.4 confirms the rapid electron transport for hydrogen evolution (Fig. 3d).^28^

**Fig. 4:**
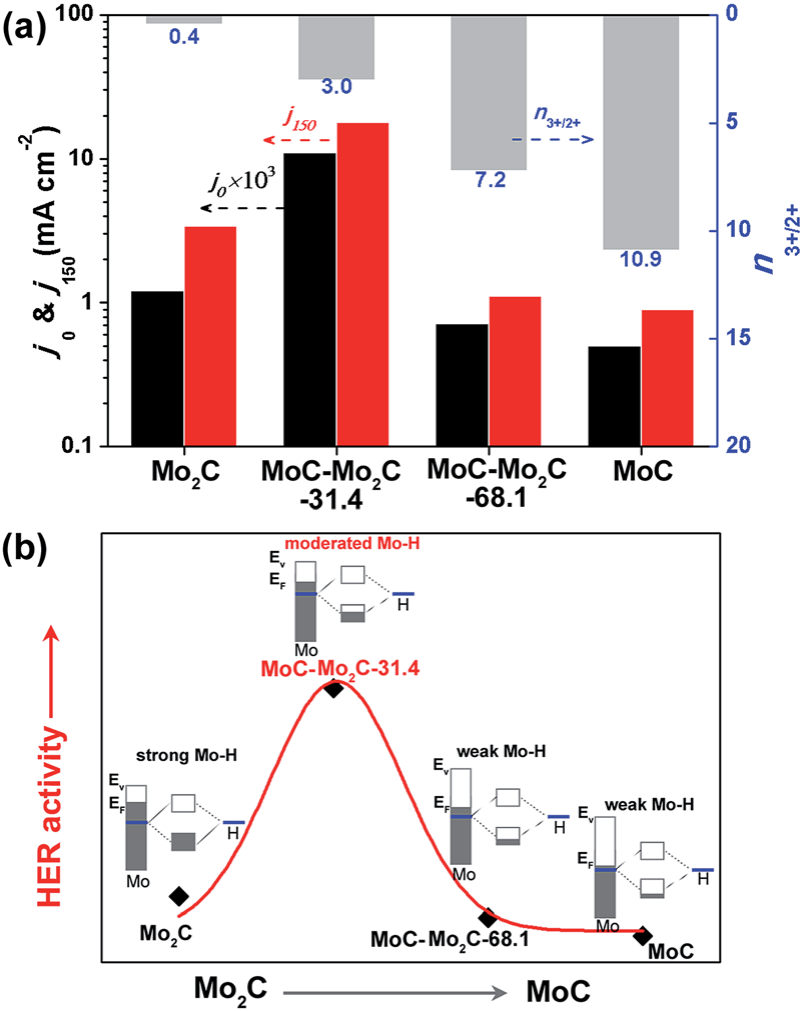
(a) *j*_0_ (obtained by extrapolating the Tafel curves to *η* = 0 mV) and *j*_150_ (current density at *η* = 150 mV) of the above MoC_*x*_, which are associated with the ratio of surface Mo^3+^/Mo^2+^ determined through XPS analysis. (b) Schematic illustration of the HER activity relying on the electron density of Mo in a series of MoC_*x*_ electrocatalysts.

It has been reported that the HER activity of MoC_*x*_ depends on the active Mo^2+^ and Mo^3+^ centres exposed on the catalyst surface,^17^,^38^ which present various Mo–H resulting from the different electron densities of Mo.^16^ As displayed in Fig. 4a, the HER activities of Mo_2_C, MoC–Mo_2_C-31.4, MoC–Mo_2_C-68.1 and MoC are dependent on the variation of the ratio of active Mo^3+^/Mo^2+^ (*n*_3+/2+_) on the surface, featured by the both of the current densities at *η* = 0 (*j*_0_) and 150 mV (*j*_150_). With a higher *n*_3+/2+_ of 3.0 in comparison with Mo_2_C (*n*_3+/2+_ = 0.4), MoC–Mo_2_C-31.4 shows an obviously improved activity, which suggests that the enriched Mo^3+^ species with fewer electrons benefits HER. Furthermore, the narrowed valance-band (VB) distribution of MoC–Mo_2_C-31.4 (Fig. S9†) indicates the lower electron density around the Fermi level (*E*_F_) than that of Mo_2_C. Regarding the strong hydrogen binding on Mo_2_C and the consequently restricted H_ads_ desorption, the decreased electron density in the MoC–Mo_2_C-31.4 HNWs would reduce the strength of Mo–H towards the promoted H_ads_ desorption and thus remarkably improved the HER activity (Fig. 4b). Moreover, with *n*_3+/2+_ increased to 7.2 and 10.9, respectively, MoC–Mo_2_C-68.1 and MoC display further reduced electron density around *E*_F_ (Fig. S9†) and decreased HER activity in comparison with MoC–Mo_2_C-31.4 (Fig. 4a). Their higher Tafel slopes (53 mV dec^–1^ for MoC–Mo_2_C-68.1, and 62 mV dec^–1^ for MoC) suggest that the limitation of the Volmer step becomes more obvious, because of weak hydrogen-binding involving less electron donated by Mo (Fig. 4b). It's reasonable that the high activity of the MoC–Mo_2_C-31.4 HNWs is ascribed to the optimized electronic properties of the MoC–Mo_2_C interfaces with a well-defined composition.

The HER activity of MoC–Mo_2_C-31.4 is superior to most of the carbide-based HER electrocatalysts that have ever been reported in acidic electrolytes (Table S4†). The *η*_10_ of 126 mV delivered by MoC–Mo_2_C-31.4 is obviously lower than that of the reported nanoporous Mo_2_C NWs (130 mV),^21^ MoCN NPs (140 mV),^17^ MoC_*x*_ nano-octahedrons (142 mV),^48^ Mo_2_C nanotubes (172 mV),^26^ and even that of supported MoC_*x*_ (Mo_2_C/CNT-GR: 130 mV;^27^ Mo_2_C/N-doped CNT: 147 mV;^49^ Mo_2_C/RGO: 150 mV;^29^ Mo_2_C/CNT: 152 mV 28). To the best of our knowledge, the lower *η*_10_ than our MoC–Mo_2_C-31.4 has been only achieved on GR or N-doped carbon encapsulated Mo_2_C NPs, which require precise control over the N-doping and thickness of the carbon shells.^38^,^45^ In regard of the high mass loading of the previously reported electrocatalysts (0.21–2.0 mg cm^–2^), the remarkably low one in this work (0.14 mg cm^–2^) strongly supports the superior activity of the MoC–Mo_2_C-31.4 HNWs. Meanwhile, the fast HER kinetics of MoC–Mo_2_C-31.4 are also confirmed by its low *η*_onset_ (38 mV) and Tafel slope (43 mV dec^–1^), which outperform most of the reported MoC_*x*_ (Table S4†). Furthermore, the HER performance of the MoC–Mo_2_C-31.4 HNWs is among the best reported when compared with many representative noble-metal free electrocatalysts, *e.g.*, transition-metals and their carbides, nitrides, chalcogenides and phosphides (Table S4†).

**Fig. 5:**
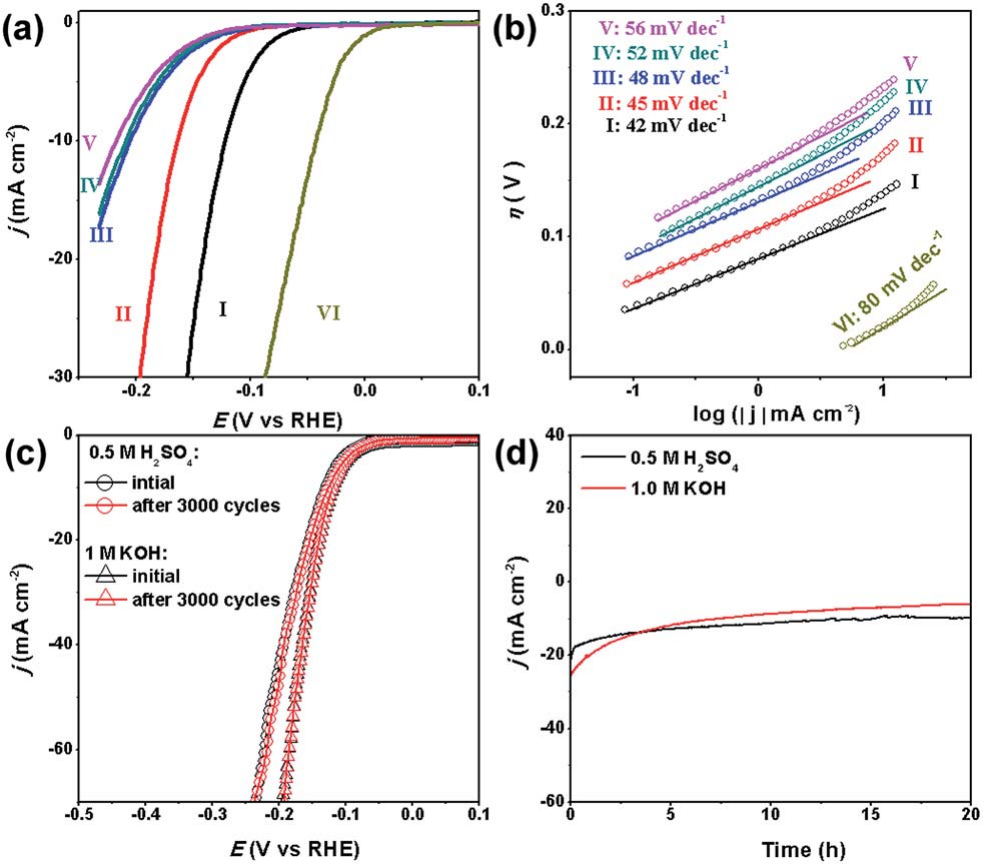
(a) Polarization curves and (b) Tafel plots for the HER on modified GCEs comprising (I) MoC–Mo_2_C-31.4, (II) Mo_2_C, (III) MoC–Mo_2_C-68.1, (IV) MoC–Mo_2_C-30 (mixed), (V) MoC, and (VI) commercial Pt/C in 1.0 M KOH. (c) Stability of the MoC/Mo_2_C-31.4 modified electrodes with an initial polarization curve and after 3000 cycles in 0.5 M H_2_SO_4_ and 1.0 M KOH, and (d) the long-term durability tests at *η* = 190 mV.

Our MoC–Mo_2_C-31.4 HNWs are also active for the HER in basic solution (1.0 M KOH), showing the best activity and kinetics in comparison with Mo_2_C, MoC, MoC–Mo_2_C-68.1 and MoC–Mo_2_C-30 (mixed) (Fig. 5a and b). This shows good consistency with its high *j*_0_, high *C*_dl_ and low *R*_ct_ (Table S5, Fig. S10 and S11†). Obviously, the synergy between MoC and Mo_2_C also promotes the HER performance in a basic electrolyte due to the optimized electronic properties of the Mo species. The *η*_10_ of 120 mV, *η*_onset_ of 33 mV and Tafel slope of 42 mV dec^–1^, observed for MoC–Mo_2_C-31.4, verify the outstanding activity performing among the best of the current MoC_*x*_ materials,^18^,^20^,^23^,^25^,^26^,^34^,^45^,^49^ and other noble-metal free electrocatalysts (Table S6†).

Interestingly, the activity of our MoC_*x*_ NWs in basic electrolyte is slightly higher than that in acidic solution. Similar situations have been observed with Mo_2_C@N-doped carbon,^30^ MoP,^50^ Mo_2_C NPs.^18^ This can be explained by the fact that the surface oxidized species on MoC–Mo_2_C can be dissolved by KOH, exposing rich active-sites for the HER (Fig. S12†).

Another important criterion for a good electrocatalyst is its high durability. Herein, the long-term stability of MoC–Mo_2_C-31.4 HNWs and the ability to continuously catalyse the generation of H_2_ were examined through continuous cycling for 3000 cycles and chronoamperometry in both 0.5 M H_2_SO_4_ and 1.0 M KOH. At the end of the cycling procedure, the catalyst affords similar *j*–*V* curves to the initial cycle with negligible loss of the cathodic current (Fig. 5c), confirming the satisfactory durability in both acidic and basic electrolytes. When further evaluated through prolonged electrolysis at a fixed potential (Fig. 5d), MoC–Mo_2_C-31.4 exhibited a catalytic current which remained at around 20 mA cm^–2^ for over 20 hours in 0.5 M H_2_SO_4_. However, the current in 1.0 M KOH slightly decreased.

## Conclusions

In summary, we have reported the facile fabrication of MoC–Mo_2_C HNWs *via* the controlled carbonization of MoO_*x*_–amine. This strategy presents significance in regulating the crystalline structure, composition and electronic properties toward efficient HER. Showing an optimized electron density on the carbide surface, the MoC–Mo_2_C-31.4 HNWs exhibit high activity and good stability in both acidic and basic solutions. This work will open up new opportunities to develop high-performance electrocatalysts *via* rational engineering of nanostructures and interfaces.

## Experimental section

### Catalyst preparation

Improved from our previous reports,^51^,^52^ MoAn-4.0 and MoAn-3.5 NWs were typically synthesized as follows: 2.48 g of ammonium heptamolybdate tetrahydrate ((NH_4_)_6_Mo_7_O_24_·4H_2_O) was dissolved in 40 mL of water consisting of 3.28 mL of Aniline. Then, 1 M HCl aqueous solution was added to adjust the pH level to 4.0 for generating MoAn-4.0, and 3.5 for achieving MoAn-3.5, respectively. After reaction at 50 °C for 4 hour in an oil bath, the products were filtered and thoroughly washed with ethanol, and then dried at 50 °C overnight. MoMeAn-4.0 NWs were prepared through a similar process to that of MoAn, replacing the aniline with 3.83 g of *p*-methylaniline.

The as-obtained MoO_*x*_-based hybrids (MoAn-4.0, MoAn-3.5 and MoMeAn-4.0) were transferred into a tube furnace and kept under an Ar flow for 4.0 h in order to remove air before heating. Then, the sample was heated to a target temperature and held for 5 h. The details for carbonization are listed in Table S1.†

### Physical measurements

SEM and TEM investigations were undertaken on a ZEISS ULTRA55 and a JEOL JEM 2100F, respectively. XRD analysis was performed on a Bruker D8 diffractometer using Cu Kα radiation (*λ* = 1.54056 Å). XPS was processed on a Perkin-Elmer PHI X-tool, using C 1s (B. E. = 284.6 eV) as a reference. TGA/DSC was tested on a NETZSCH STA449F3 under an air flow. FT-IR spectra were collected with a Nicolet 6700 FTIR spectrometer. The composition of the NWs was determined using ICP (for Mo), CHN elemental analysis using a Vario EL Elementar (for C, H and N) and an internal standard quantification in XRD (for the ratio of MoC/Mo_2_C). N_2_ adsorption–desorption isotherms were recorded on an automatic gas adsorption analyzer (Quantachrome Autosorb-iQ-MP). Raman spectra were recorded on a Raman spectrometer (Horiba), with an excitation laser wavelength of 632.81 nm.

### Electrochemical measurements

The MoC_*x*_ electrocatalysts were loaded onto GCEs for testing in 0.5 M H_2_SO_4_ and 1.0 M KOH solutions using a typical three-electrode setup. Typically, 4 mg of catalyst and 40.0 μL of 5 wt% Nafion solution were dispersed in 1 mL of 4 : 1 v/v water/ethanol through at least 30 min of sonication to form a homogeneous ink. For the test in 1.0 M KOH, 10 μL of polyvinylidene fluoride (5 wt%) was further added into the above ink. Then 2.5 μL of catalyst ink was loaded onto a GCE of 3 mm in diameter. Linear sweep voltammetry (LSV) was conducted with the scan rate of 2 mV s^–1^ in 0.5 mol L^–1^ H_2_SO_4_ or 1.0 M KOH on a potentiostat of CHI760 (CH Instruments), using a saturated calomel electrode as the reference electrode, and a graphite electrode as the counter electrode. All of the potentials reported in our manuscript were referenced to a reversible hydrogen electrode (RHE) by adding a value of (0.241 + 0.059 pH) V. AC impedance measurements were carried out in the same configuration at *η* = 200 mV from 0.01 to 1 000 000 Hz and an amplitude of 5 mV.

## Supplementary Material

Supplementary information
