# Supplementary material for: Heteronanowires of MoC–Mo2C as efficient electrocatalysts for hydrogen evolution reaction
Source: Chem Sci. 2016 Feb 12;7(5):3399–405. doi: 10.1039/c6sc00077k (PMC6007147; doi:10.1039/c6sc00077k)
Supplement: Supplementary file 1 [file SC-007-C6SC00077K-s001.pdf]

Electronic Supplementary Material (ESI) for Chemical Science.

This journal is © The Royal Society of Chemistry

Heteronanowires of MoC-Mo_2C as Efficient Electrocatalysts for Hydrogen Evolution Reaction

Huanlei Lin,[a] Zhangping Shi,[b] Sina He,[a] Xiang Yu,[a,c] Sinong Wang,[b] Qingsheng Gao*,[a] and Yi Tang*,[b]

[a] Department of Chemistry, Jinan University, Guangzhou 510632, China. E-mail: tqsgao@jnu.edu.cn

[b] Department of Chemistry, Shanghai Key Laboratory of Molecular Catalysis and Innovative Materials, Laboratory of Advanced Materials and Collaborative Innovation Center of Chemistry for Energy Materials, Fudan University, Shanghai 200433, China. E-mail: yitang@fudan.edu.cn

[c] Analytic and Testing Centre, Jinan University, Guangzhou 510632, China

Table S1 Details for the controlled synthesis of MoC_x from MoO_x-amine hybrids.

products	precursors	temperature (^oC)	ramping rate (^oC min^{-1})	Ar flow rate (mL min^{-1})
Mo_2C	MoAn-4.0	775	2	200
MoC	MoMeAn-4.0	700	5	200
MoC-Mo_2C-31.4	MoAn-3.5	775	2	200
MoC-Mo_2C-68.1	MoMeAn-4.0	775	5	200

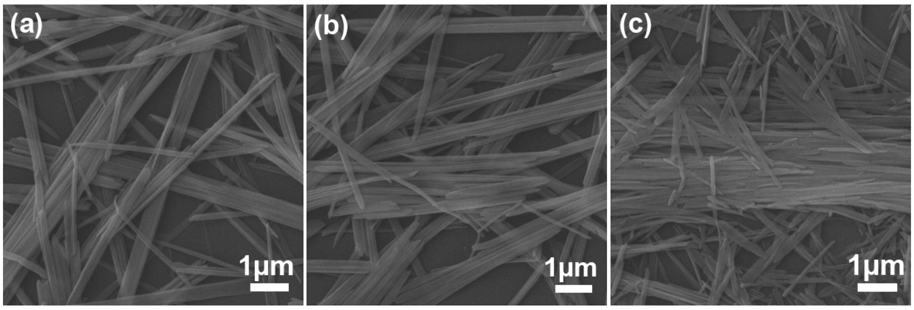

Fig. S1 SEM images of the (a) MoAn-4.0; (b) MoAn-3.5; (c) MoMeAn-4.0 hybrid NWs.

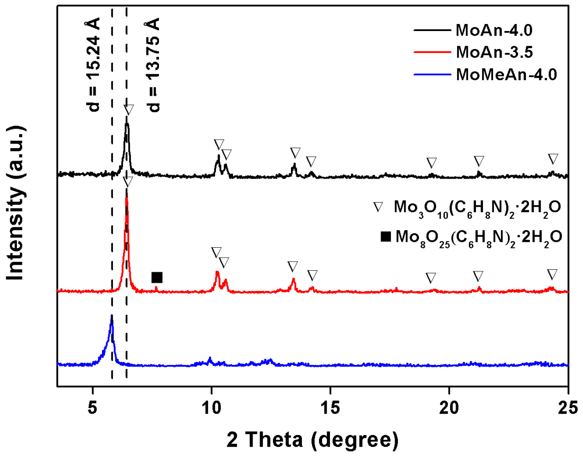

Fig. S2 XRD patterns of the MoAn-4.0; MoAn-3.5 and MoMeAn-4.0 hybrid NWs.

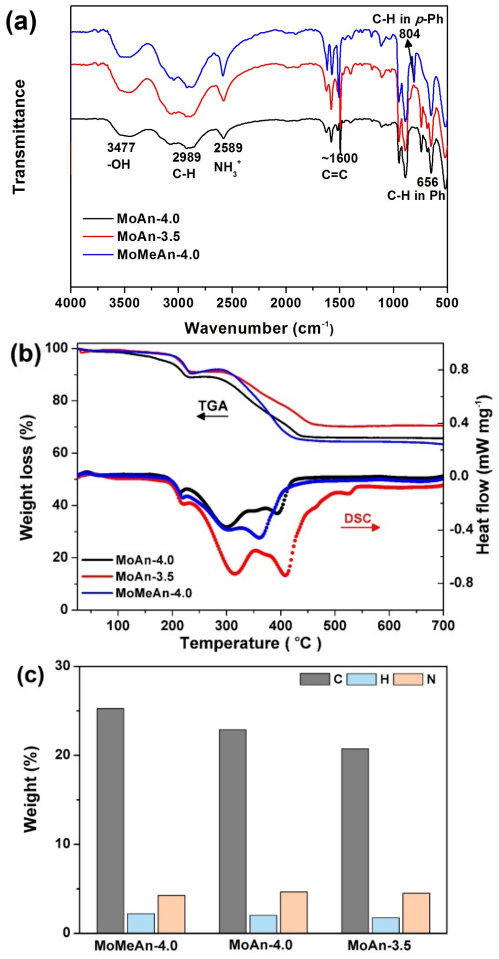

Fig. S3 (a) FT-IR spectra, (b) TGA/DSC curves, and (c) CHN elemental analysis of MoAn-4.0, MoAn-3.5 and MoMeAn-4.0.

Table S2 Composition of as-obtained MoC_x determined by the combined CHN elemental analysis, ICP-AES and XRD.

Samples	Mo_2C (wt%)	MoC (wt%)	C (wt%)
MoC-Mo_2C-31.4	67.0	31.4	1.6
Mo_2C	96.8	/	3.2
MoC-Mo_2C-68.1	28.3	68.1	3.6
MoC	/	95.7	4.3

Table S3 Fitting parameters (peak position, peak area and species percentage) for both Mo $3d_{5/2}$ and Mo $3d_{3/2}$ spectra taken on Mo_2C, MoC, MoC-Mo_2C-31.4 and MoC-Mo_2C-68.1.

Samples	species	B. E. (eV)		area		Mo^{3+}/Mo^{2+}	Mo^{3+} and Mo^{2+} (%)
		$3d_{5/2}$	$3d_{3/2}$	$3d_{5/2}$	$3d_{3/2}$		
Mo_2C	Mo^{2+}	228.2	231.3	5014	3512	0.4	35.4
	Mo^{3+}	228.8	231.9	1750	1310		
	Mo^{4+}	229.9	233.0	3651	2203		
	Mo^{6+}	232.5	235.6	9998	5790		
MoC	Mo^{2+}	228.2	231.3	1824	1291	10.9	50.2
	Mo^{3+}	228.8	231.9	20682	13598		
	Mo^{4+}	230.0	233.3	4959	3189		
	Mo^{6+}	232.5	235.6	18012	11184		
MoC-Mo_2C-31.4	Mo^{2+}	228.3	231.3	5082	3301	3.0	50.7
	Mo^{3+}	228.7	231.8	15459	10013		
	Mo^{4+}	229.9	233.1	7986	6228		
	Mo^{6+}	232.5	235.5	11510	7084		
MoC-Mo_2C-68.1	Mo^{2+}	228.2	231.3	1801	1291	7.2	40.8
	Mo^{3+}	228.7	231.9	13310	8398		
	Mo^{4+}	230.0	233.1	3612	2401		
	Mo^{6+}	232.5	235.6	17980	11729		

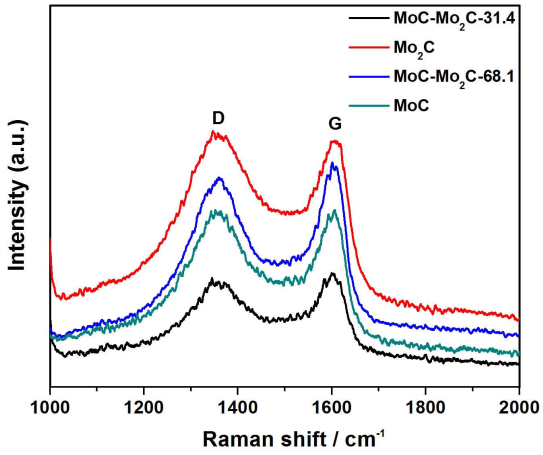

Fig. S4 Raman spectra of MoC-Mo_2C-31.4, Mo_2C, MoC-Mo_2C-68.1 and MoC.

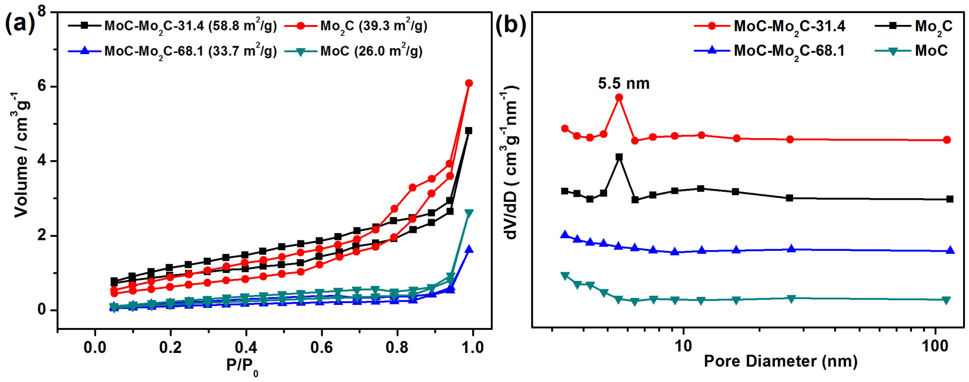

Fig. S5 (a) N_2-sorption isotherms and (b) pore size distribution plots of MoC-Mo_2C-31.4, Mo_2C, MoC-Mo_2C-68.1 and MoC.

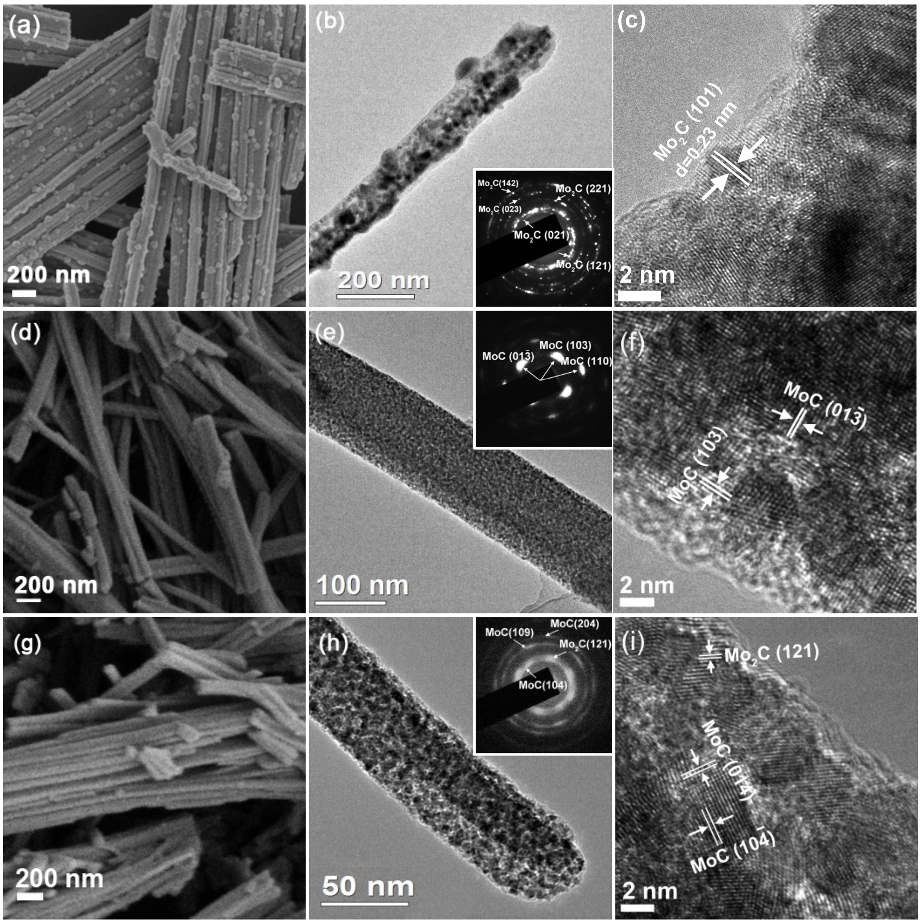

Fig. S6 (a, d, g) SEM, (b, e, h) TEM and (c, f, i) HR-TEM images of (a, b, c) Mo_2C, (d, e, f) MoC and (g, h, i) MoC-Mo_2C-68.1. Insets of b, e and h are the SAED patterns obtained on the corresponding single nanowire.

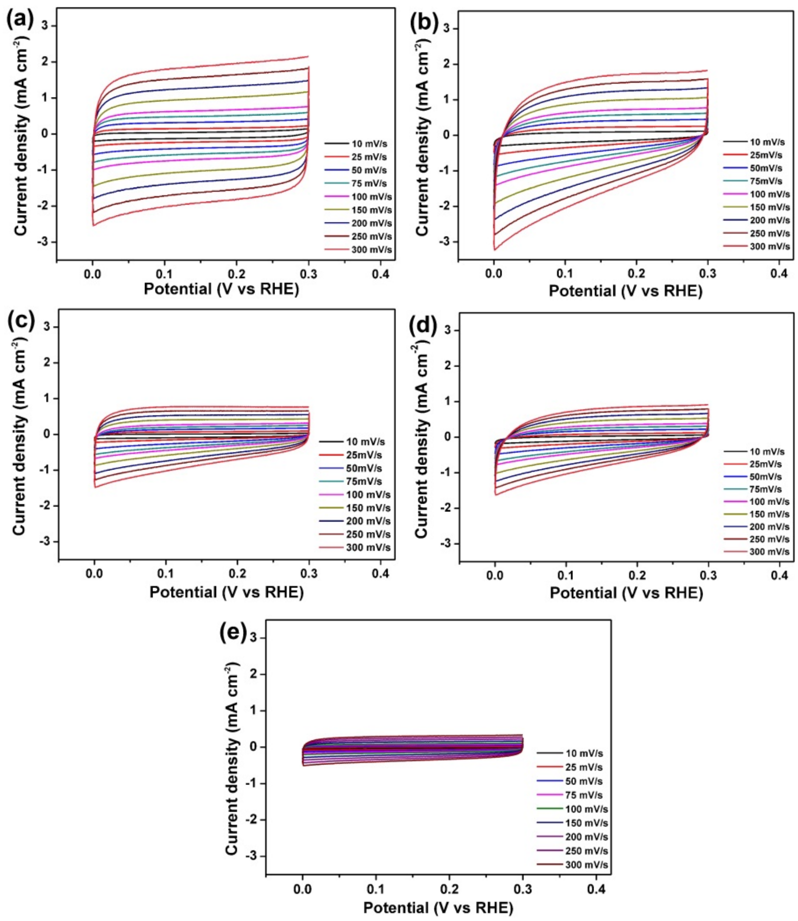

Fig. S7 Cyclic voltammograms of (a) MoC-Mo_2C-31.4, (b) Mo_2C, (c) MoC-Mo_2C-68.1, (d) MoC-Mo_2C-30 (mixed), with various scan rates in 0.5 M H_2SO_4.

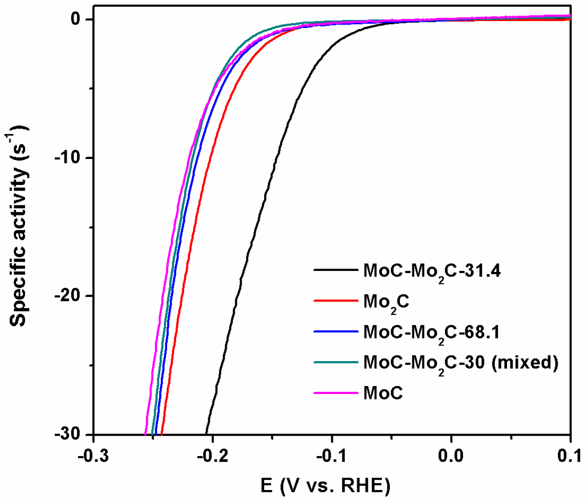

Fig. S8 The specific activity of various MoC_x electrocatalysts in 0.5 M H_2SO_4. Data is obtained by dividing the current density by their corresponding C_{dl}, and further 0.3 V (the voltage range used in CV).

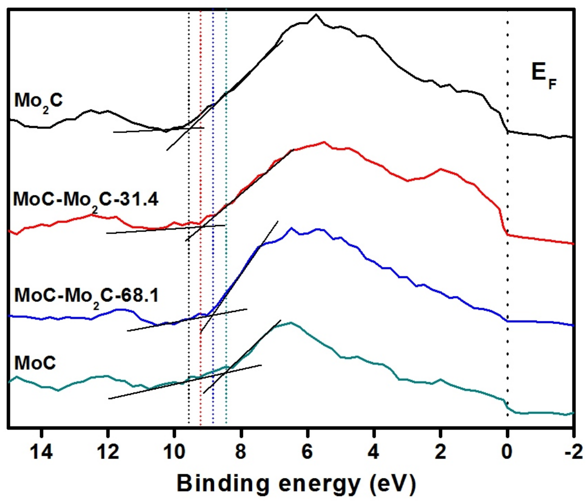

Fig. S9 (a) XPS spectra of valence bands for the as-obtained MoC_x NWs.

Table S4 Comparison of HER performance in acid media for MoC-Mo_2C-31.4 HNWs with other electrocatalysts.

catalysts	loading (mg cm^{-2})	*j*(mA cm^{-2})	*η*(mV)	Tafel slope (mV dec^{-1})	ref.
MoC-Mo_2C-31.4	0.14	1	46	43	This work
		10	126		
MoC_x octahedrons	0.8	1	87	53	*Nat. Commun.* **2015**, *6*, 6512.
		10	142		
Mo_2C/CNT	2.0	1	63	65	*Energy Environ. Sci.* **2013**, *6*, 943.
		10	152		
np-Mo_2C NWs	0.21	10	130	53	*Energy Environ. Sci.* **2014**, *7*, 387.
Mo_2C microparticles	1.4-2.5	20	~225	55-56	*Angew. Chem. Int. Ed.* **2012**, *51*, 12703.
MoCN	0.4	10	140	46	*J. Am. Chem. Soc.* **2015**, *137*, 110.
Mo_2C nanotubes	0.75	10	172	62	*Angew. Chem. Int. Ed.* **2015**, *54*, 15395.
Mo_2C@NC	0.28	10	124	60	*Angew. Chem. Int. Ed.* **2015**, *54*, 37.
Mo_2C@C	0.25	10	78	41	*Angew. Chem. Int. Ed.* **2015**, *54*, 12723.
Mo_2C/CNT-GR	0.65-0.67	10	130	58	*ACS Nano* **2014**, *8*, 5164.
Mo_2C/RGO	0.285	10	130	57.3	*Chem. Commun.* **2014**, *50*, 13135.
Double-gyroid MoS_2	0.022	6.74	200	43-47	*Nat. Mater.* **2012**, *11*, 963.
Edge-terminated MoS_2	0.28	10	149	49	*Nat. Commun.* **2015**, *6*, 7493.
MoS_2@N-doped carbon	1.0	10	165	55	*Angew. Chem. Int. Ed.* **2015**, *54*, 7395.
Hierarchical MoS_2	1.0	10	167	70	*Adv. Mater.* **2015**, *27*, 7426.
$NiMoN_x$/C	0.25	2	170	36	*Angew. Chem. Int. Ed.* **2012**, *51*, 6131.
MoN nanosheets	0.28	10	~200	90	*Chem. Sci.***2014**, *5*, 4615.
MoP NPs	0.36	10	125	54	*Adv. Mater.* **2014**, *26*, 5702.
WC-CNTs	/	10	145	72	*ACS Nano* **2015**, *9*, 5125.
Fe-WCN particles	0.4	10	220	47.1	*Angew. Chem. Int. Ed.* **2013**, *52*, 13638.
P-WN/rGO	0.337	10	85	54	*Angew. Chem. Int. Ed.* **2015**, *54*, 6325.
Ni_2P hollow particles	1	10	116	46	*J. Am. Chem. Soc. **2013**, 135, 9267.*
CoP/CNT	0.285	10	122	54	*Angew. Chem. Int. Ed.* **2014**, *126*, 6828.
Cu_3P NWs/CF	15.2	10	143	67	*J. Am. Chem. Soc.* **2014**, *136*, 7587.
Co/N-rich CNTs	0.28	10	260	69	*Angew. Chem. Int. Ed.* **2014**, *53*, 4372.

Table S5 Summary of HER activities of MoC-Mo_2C-31.4, Mo_2C, MoC-Mo_2C-68.1, MoC-Mo_2C-30 (mixed), and MoC in 1.0 M KOH.

samples	η_{10} (mV)	η_{onset} (mV)	Tafel slope (mV dec^{-1})	R_{ct}[a] (Ω)	C_{dl}[b] (mF cm^{-2})	j_0[c] (mA cm^{-2})
MoC-Mo_2C-31.4	120	33	42	16.9	6.98	1.3×10^{-2}
Mo_2C	164	51	45	57.2	5.78	$1.2*10^{-3}$
MoC-Mo_2C-68.1	206	82	48	101.7	5.28	8.7×10^{-4}
MoC-Mo_2C-30 (mixed)	212	95	52	105	4.01	4.1×10^{-4}
MoC	221	101	56	135	2.50	5.8×10^{-4}

[a] Data was measured at η = 200 mV; [b] Data was calculated according the CV results (Fig. S10 in SI); [c] Exchange current densities (j_0) were obtained from Tafel curves by using extrapolation methods.

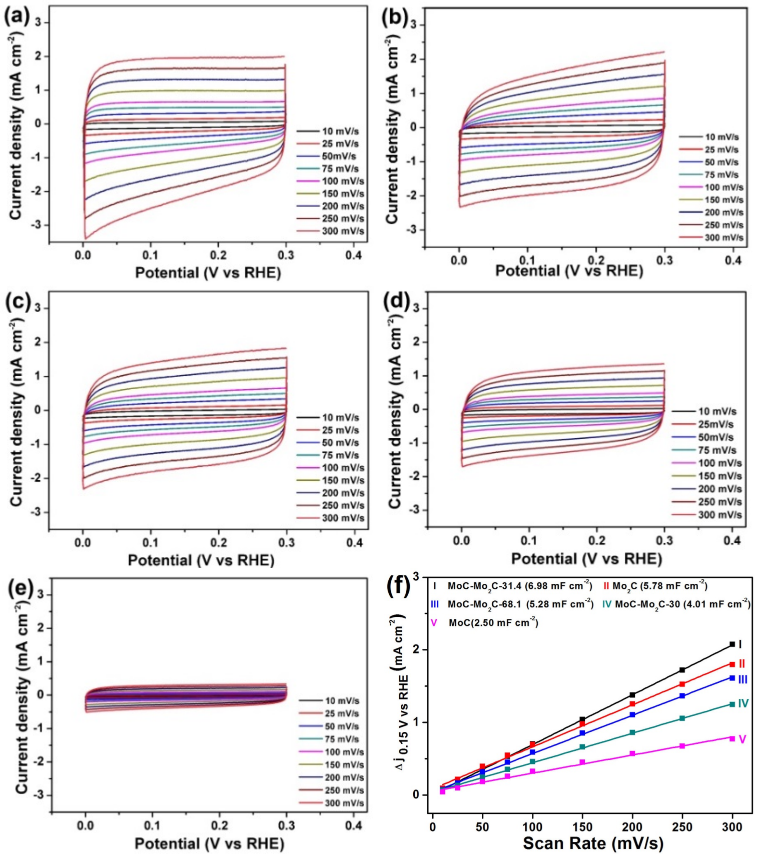

Fig. S10 Cyclic voltammograms of (a) MoC-Mo_2C-31.4, (b) Mo_2C, (c) MoC-Mo_2C-68.1, (d) MoC-Mo_2C-30 (mixed) and (e) MoC. (f) C_{dl} of above MoC_x determined by the slope as the capacitive current density ($\Delta j = (j_a+j_c)/2$) was plotted as a function of scan rate in 1.0 M KOH.

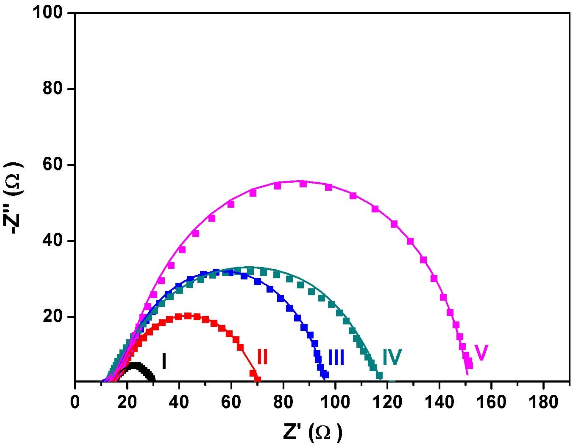

Fig. S11 Electrochemical impedance spectroscopy (EIS) of the as-obtained (I) MoC-Mo_2C-31.4, (II) Mo_2C, (III) MoC-Mo_2C-68.1, (IV) MoC-Mo_2C-30 (mixed), and (V) MoC in 1.0 M KOH at η = 200 mV.

Table S6 Comparison of HER performance in basic media for MoC-Mo_2C-31.4 HNWs with other electrocatalysts.

catalysts	loading (mg cm^{-2})	*j* (mA cm^{-2})	η（mV）	Tafel slope (mV dec^{-1})	ref.
MoC-Mo_2C-31.4	0.14	1<br>10	42<br>120	42	This work
MoC_x octahedrons	0.8	1<br>10	92<br>151	59	*Nat. Commun.* 2015, 6, 6512.
Mo_2C microparticles	0.8-2.3	20	210-240	54-59	*Angew. Chem. Int. Ed.* **2012**, *51*, 12703.
Ni-Mo_2C nanorods	0.43	10	ca. 140	49	*Appl. Catal., B* **2014**, *154*, 232.
Mo_2C nanotubes	0.75	10	112	55	*Angew. Chem. Int. Ed.* **2015**, *54*, 15395.
Mo_2C@NC	0.28	10	60	/	*Angew. Chem. Int. Ed.* **2015**, *54*, 37
Mo_2C@C	0.25	10	78	41	*Angew. Chem. Int. Ed.* **2015**, *54*, 14723.
WC-CNTs	/	10	165	72	*ACS Nano* **2015**, *9*, 5125.
MoS_2 nanosheet arrays	/	10	190	100	*Electrochim. Acta* **2015**, *168*, 256.
MoP	0.86	10	130	48	*Energy Environ. Sci.* **2014**, *7*, 2624.
Ni-Mo nanopowder	1	10	~80[a]	/	*ACS Catal.* **2013**, *3*, 166.
Co@Co-oxo/hydoxo phosphate	/	2	385	140	*Nat. Mater.* **2012**, *11*, 802.
CoP NW arrays	0.92	1<br>10	115<br>209	129	*J. Am. Chem. Soc.* **2014**, *136*, 7587.
NiS_2 nanosheets	4.1	10	150	69	*Electrochimica Acta* **2015**, *153*, 508.
Ni_3S_2/nickel foam	1.6	10	223	/	*J. Am. Chem. Soc.* **2015**, *137*, 14023.
NiP_2 nanosheet	4.3	10	102	64	*Nanoscale* **2014**, *6*, 13440.
WP NW arrays	2.0	10	150	102	*ACS Appl. Mater. Interfaces* **2014**, *6*, 21874.

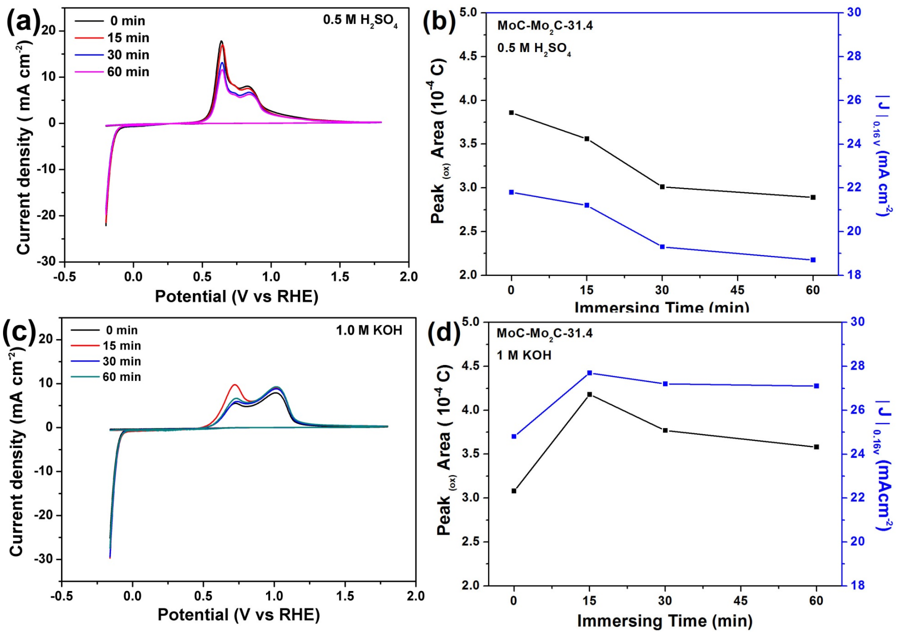

Fig. S12 (a) CVs of the MoC-Mo_2C-31.4 HNWs in the range of -0.16 to 1.90 V (vs. RHE) in 0.5 M H_2SO_4. (b) Oxidation peak area and the current density with different immersing time in 0.5 M H_2SO_4. (c) CVs of the MoC/Mo_2C-31.4 in the range of -0.16 to 1.90 V (vs. RHE) in the basic solution. (d) Oxidation peak area and the current density with different immersing time in 1.0 M KOH.

Obviously, MoC-Mo_2C-31.4 can be oxidized by scanning the potential from -0.16 to 1.8 V (vs. RHE), and the integral peak area for anodic current can be used to visualize the amount of active Mo^{2+} and Mo^{3+}. Meanwhile, the *j* at -0.16 V (vs. RHE) detected before the positive scanning to oxidize active Mo^{2+} and Mo^{3+} denotes the HER activity. Immersed with 0.5 M H_2SO_4, the area of anodic peaks and the *j* at -0.16 V (vs. RHE) are both slightly decreased with treating time, which may be related with the occupation of active-sites by bulky SO_4^{2-}. In comparison, the area of anodic peaks is obviously increased with an increased time for KOH treatment, accompanied with the improved *j* for HER at -0.16 V (vs. RHE). This should be ascribed to exposed active carbide surface after removing surface oxides by KOH. Thus, our MoC-Mo_2C-31.4 exhibits a slightly higher HER activity in basic electrolyte in comparison with that in acidic solution.
